# Associations between doses of fall-risk-increasing drugs (FRIDs) and falls of hospitalized patients

**DOI:** 10.1038/s41598-023-41568-6

**Published:** 2023-09-01

**Authors:** Yu-Kai Yang, Chew-Teng Kor, Yi-Wei Sun, Hsin-Yu Wang, Yuan-Ting Yang, Sen-Yung Liu

**Affiliations:** 1 Department of Physical Medicine and Rehabilitation, Changhua Christian Hospital, 135 Nanhsiao Street, Changhua City, 50006 Taiwan, ROC; 2 Big Data Center, Changhua Christian Hospital, Changhua City, Taiwan, ROC; 3 Department of Pharmacy, Changhua Christian Hospital, Changhua City, Taiwan, ROC

**Keywords:** Health care, Medical research, Risk factors

## Abstract

Falls are a serious public health problem in the aging population because of the associated clinical and socioeconomic impact. Although previous studies have investigated fall-risk-increasing drugs (FRIDs), few studies have focused on dosage among adult inpatients. This study aimed to evaluate associations between fall risk and dosage of different FRIDs classes in hospital inpatients. Inpatients who experienced falls at medical or surgical wards of Changhua Christian Hospital from January 2017 to December 2021 were identified and matched by age, sex, and hospital ward to randomly selected controls (four per case). Anonymous patient data were extracted from the hospital medical data repository, including demographic characteristics, comorbidities, fall-risk scores, and drug prescriptions. Medication dosages were computed using the anatomical therapeutic chemical classification and the defined daily dose system of the World Health Organization. A total of 852 cases and 3408 controls were identified as eligible. Reducing the use of CNS-active medications, administering lower doses of sedative-hypnotics, prescribing sufficient dopaminergic anti-Parkinson agents, and using NSAIDs instead of opioids are imperative in preventing falls among hospitalized patients according to the findings in the study.

## Introduction

Falls are the most common adverse events among adult inpatients^1^. One in three hospital falls result in an injury and about 5% involve serious trauma^2^. In addition to physical injury, falls lead to functional decline, poor quality of life, prolonged hospital stays, and increased medical expenses^3^. Due to their clinical and socioeconomic impact, falls have become a critical health issue in the aging society^4^.

Cognitive dysfunction and visual impairment are among the major risk factors associated with falls, along with balance and gait disorders, history of previous falls, polypharmacy and fall-risk-increasing drugs (FRIDs)^5,6^. Previous studies^7,8^ have reported that FRIDs commonly include antipsychotics, anxiolytics, sedatives, antidepressants, narcotics, antiepileptics, and cardiovascular medications. Although several studies have investigated FRIDS^9,10^, few have focused on the dose of FRIDs received by adult inpatients. Therefore, we remain uncertain about whether varying dosages of fall-risk-increasing drugs (FRIDs) would lead to different probabilities of falls. It's unclear whether low dosages of FRIDs would heighten the likelihood of falling, or if low dosages can be safely administered in clinical settings while only high dosages carry risks. Our assumption is that even the consumption of low dosages of FRIDs could elevate the risk of falling.

This retrospective case–control study aimed to evaluate associations between risk of falls and dosage of different classes of FRIDs in hospital inpatients.

## Patients and methods

### Study design and population

This retrospective case–control study was conducted in Changhua Christian Hospital, Changhua, Taiwan, which admits about 330,000 patients annually. The facility employs a staff of about 5000 and has more than 1400 beds. All adult patients (aged 20 years or older) who were admitted to a medical or surgical ward and had a fall during the hospitalization period were eligible for the study. Patients with missing data were excluded. Inpatients who experienced a fall in the medical or surgical ward of Changhua Christian Hospital from January 2017 to December 2021 and were reported in the nursing adverse event reporting system (AERS) were included. Included patients were matched by age, sex, and hospital ward to controls without falls (four patients for each fall case) who were selected through systematic random sampling.

To balance the influence of confounding variables within the fall group, a propensity score was calculated for each patient, considering the patient’s age, gender, and hospital ward in a non-parsimonious multivariate logistic regression model. Subsequently, a propensity score matching analysis was performed using a 1:4 ratio, pairing was performed using the nearest neighbor technique, and individuals with equal propensity scores were required to be paired, maintaining equivalence between groups.

### Data collection

Anonymous patient data, including demographic characteristics, comorbidities, fall-risk scores, and prescriptions within 3 days before the fall (dose, type of medications and ATC [Anatomical Therapeutic Chemical Classification System] codes) were extracted from the hospital medical records repository. Prescriptions of the control group within the last 3 days before discharge were also extracted. Medications doses were calculated using the anatomical therapeutic chemical (ATC) classification and the defined daily dose (DDD) system of the World Health Organization^11^.

### Ethical considerations

The Institutional Review Board (IRB) Committee A of Changhua Christian Hospital in Taiwan (IRB Nunber: 220818) reviewed and approved the study protocol titled “Prediction of Falls Risk and Falls Risk Increase Drugs Using Big Data Analysis” via a document dated 08/31/2022. Since the study was retrospective and all subjects' private data were de-identified, the IRB declared that the requirement for signed informed consent was not necessary. All clinical investigations were conducted in accordance with the guidelines of the 2013 Declaration of Helsinki.

### Outcome assessment

The study focused on the occurrence of falls during patients’ hospital stays. These events were meticulously documented and sourced from the hospital's nursing adverse event reporting system (AERS). According to the established hospital policy, every patient incident must be exhaustively reported and accurately recorded within the specified reporting framework. This stringent approach ensures a comprehensive and accurate representation of falls as they occur.

### FRIDs classifications

Information regarding FRID exposures was extracted from the electronic medication administration records of inpatients. We identified FRIDs as defined by the Swedish National Board of Health and Welfare in conjunction with the ATC classification system. The 20 classifications of FRIDs were categorized into 3 groups: CNS-active medications (including opioids, antipsychotics, anxiolytics, hypnotics and sedatives, antidepressants, and antiepileptic), cardiovascular (including vasodilators, antihypertensives, diuretics, beta-blockers and calcium channel blockers, renin-angiotensin system inhibitors, alpha adrenergic receptor antagonists, and digitalis glycoside), and others (including anti-Parkinson drugs-dopaminergic agents and anticholinergic agents, anti-diabetes agents, NSAIDs, contact laxatives, and proton pump inhibitors). Detailed information, including the ATC codes and corresponding defined daily Doses (DDDs), is presented in Table 3.

### Other confounders

Patients' demographics and clinical characteristics were obtained from the hospital medical records repository. This information included age, sex, education status, severe illness, BMI, smoking and alcohol consumption history, physical exercise, surgical history, number of comorbidities at admission, as well as nursing assessments at admission (fall scale, fall history, Barthel index, dysphagia, visual impairment, hearing impairment, and sleep disorder). Additionally, laboratory results upon admission were recorded, including serum hemoglobin (Hb), creatinine, eGFR, white blood cell count (WBC), platelet count, potassium (K), and sodium (Na). Admission diagnosis and vital signs (systolic and diastolic blood pressure, pulse rate, respiratory rate, and body temperature) within the 24 h before the fall were also obtained.

### Statistical analysis

Continuous variables were assessed using Student’s t-tests for normally distributed data, and Mann–Whitney U tests were used to analyze abnormally-distributed data. The chi-squared test and Fisher’s exact test were performed to compare categorical variables. After adjusting for confounders, logistic regression models were used to evaluate associations between the 20 types of FRIDs and fall events. Results are presented as odds ratios (ORs) with 95% confidence intervals (CIs), and crude odds ratios (ORs) and adjusted odds ratios (aORs) of taking FRIDs. All statistical analysis was performed using SAS version 9.4 (SAS Institute Inc., Cary, NC, USA) and 2-tailed *P* values of < 0.05 were considered statistically significant.

## Results

**Figure 1:**
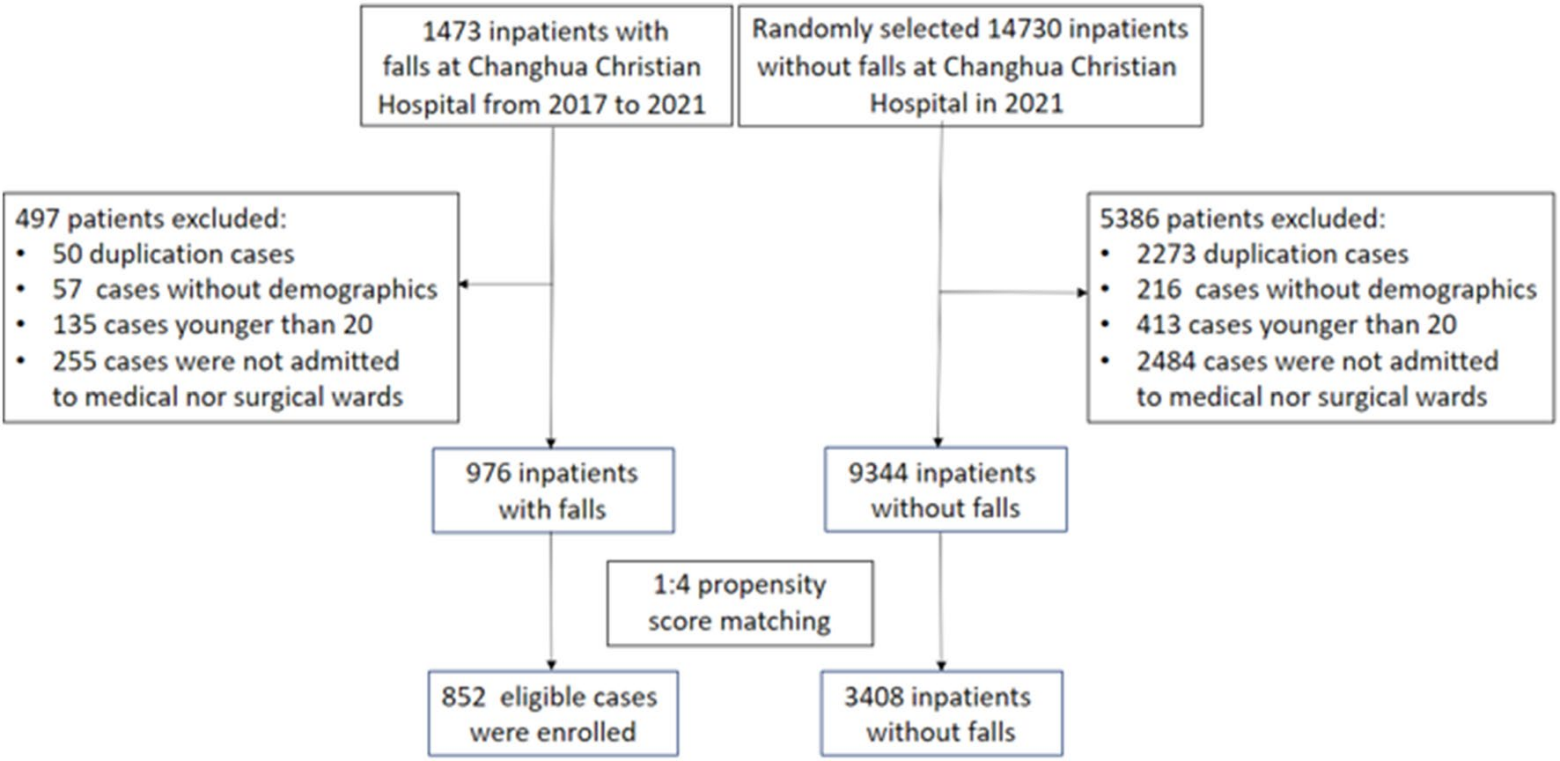
Patient flow diagram.

A total of 976 adult inpatients experienced at least one fall in a medical or surgical ward of Changhua Christian Hospital during the study period. Of these, a total of 852 inpatients were matched by age, sex and ward in a 1:4 ratio as presented in Fig. 1. Matched covariates for age, sex and ward were well balanced. The mean age of the patients was 65 years, and 59.6% were male.

**Figure 2:**
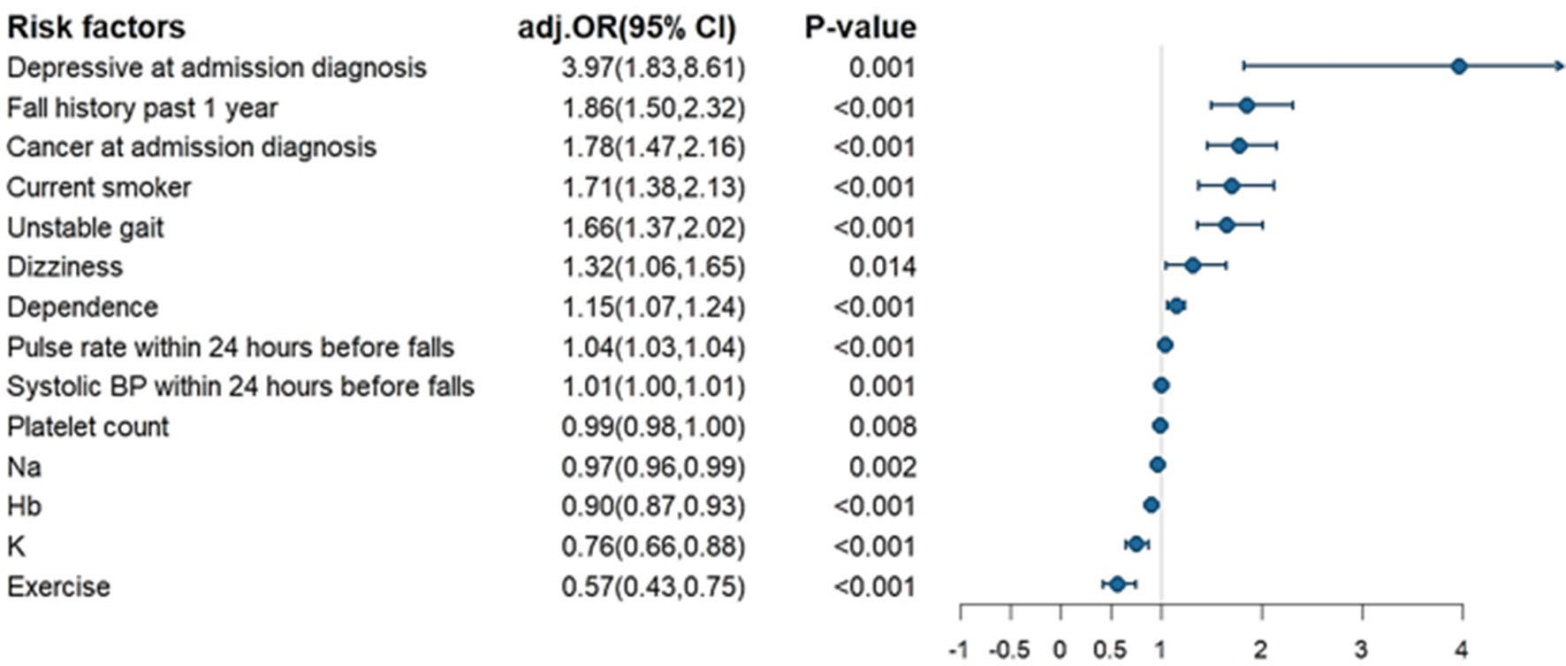
Conditional logistic regression analysis of demographic and clinical characteristics associated with falls during hospitalization.

Demographics, number of comorbidities, surgical history, fall scale scores, patient dependency (Barthel index), and clinical characteristics are shown in Table 1. Patients with falls had more severe illnesses, more comorbidities, lower education, more cigarette use, lower physical activity, higher fall scale scores, increased patient dependency, higher prevalence of dysphagia, visual impairment, and sleep disorders. Among clinical variables, patient with falls tended to have tachycardia, anemia, and hyponatremia, with a primary admitting diagnosis of cancer, depression, chronic kidney disease, stroke or cardiovascular diseases. After adjustments for confounders, patients with cancer and depression as primary admitting diagnosis, cigarette use, fall history in the past 1 year, dizziness, unsteady gait, increased patient dependency, tachycardia, anemia, hyponatremia, and hypokalemia were especially prone to falls (Fig. 2). Physical activity was the only protective factor associated with falls.

**Table 1:** Demographic and clinical variables of 852 fall cases and 3408 non-fall controls among inpatients.

Sample size	Non-fall cases	Fall cases	*P* value	Crude OR (95% CI)
	3408	852		
Age	64.95 ± 14.99	65.05 ± 14.98	0.86	1.00 (0.96, 1.06)
Age ≥ 65	1827 (53.6%)	460 (54%)	0.84	1.02 (0.87, 1.18)
Male	2033 (59.7%)	506 (59.4%)	0.89	0.99 (0.85, 1.15)
Severe illness	304 (8.9%)	148 (17.4%)	< 0.001	2.15 (1.74, 2.66)
Body mass index	24.66 ± 4.62	23.68 ± 4.9	< 0.001	0.95 (0.94, 0.97)
Number of comorbidities at admission				
None	951 (27.9%)	168 (19.7%)	< 0.001	1 (reference)
1	1118 (32.8%)	250 (29.3%)	0.03	1.27 (1.02, 1.57)
2	787 (23.1%)	250 (29.3%)	< 0.001	1.80 (1.45, 2.23)
≥ 3	552 (16.2%)	184 (21.6%)	< 0.001	1.89 (1.49, 2.38)
Current smoker	551 (16.2%)	167 (19.6%)	0.02	1.26 (1.04, 1.53)
Alcohol	92 (2.7%)	30 (3.5%)	0.20	1.32 (0.87, 2.00)
Exercise	688 (20.2%)	74 (8.7%)	< 0.001	0.38 (0.29, 0.48)
Surgical history	2362 (69.3%)	616 (72.3%)	0.09	1.16 (0.98, 1.37)
Fall scale scores at admission				
Unsteady gait	928 (27.2%)	444 (52.1%)	< 0.001	2.91 (2.49, 3.39)
Dizziness	405 (11.9%)	188 (22.1%)	< 0.001	2.10 (1.73, 2.54)
Muscle weakness	486 (14.3%)	212 (24.9%)	< 0.001	1.99 (1.66, 2.39)
Mobility aids use	848 (24.9%)	396 (46.5%)	< 0.001	2.62 (2.24, 3.06)
Fall history in past 1 year	356 (10.4%)	223 (26.2%)	< 0.001	3.04 (2.52, 3.67)
Dysphagia	257 (7.5%)	85 (10%)	0.02	1.36 (1.05, 1.76)
Physiological evaluation				
Visual impairment	624 (18.3%)	206 (24.2%)	< 0.001	1.42 (1.19, 1.70)
Hearing impairment	365 (10.7%)	104 (12.2%)	0.21	1.16 (0.92, 1.46)
Sleep disorder	68 (2%)	33 (3.9%)	0.001	1.98 (1.30, 3.02)
Barthel index	77.24 ± 31.25	69.51 ± 29.72	< 0.001	0.93 (0.91, 0.95)
Dependence				
No dependence (Barthel index = 100)	1720 (50.5%)	243 (28.5%)	< 0.001	1 (reference)
Slight dependence (91–99)	64 (1.9%)	20 (2.3%)	0.003	2.21 (1.32, 3.72)
Moderate dependence (61–90)	707 (20.7%)	263 (30.9%)	< 0.001	2.63 (2.17, 3.20)
Severe dependence (21–60)	577 (16.9%)	246 (28.9%)	< 0.001	2.52 (2.09, 3.03)
Total dependence (0–20)	340 (10%)	80 (9.4%)		
Laboratory data at admission				
Hemoglobin	12.59 ± 2.38	11.32 ± 2.52	< 0.001	0.81 (0.79, 0.84)
Creatinine	1.37 ± 1.88	1.69 ± 2.49	0.001	1.07 (1.03, 1.10)
eGFR	77.69 ± 36.1	73.85 ± 41.95	0.014	0.97 (0.95, 0.99)
WBC	9.19 ± 9.74	9.75 ± 10.66	0.163	1.05 (0.98, 1.12)
Platelet	227.94 ± 94.24	222.83 ± 118.45	0.24	0.99 (0.99, 1.01)
K	3.92 ± 0.53	3.86 ± 0.64	0.02	0.82 (0.71, 0.95)
Na	136.55 ± 4.88	134.7 ± 4.89	< 0.001	0.93 (0.91, 0.94)
Admission diagnosis				
Anemia	187 (5.5%)	64 (7.5%)	0.03	1.4 (1.04, 1.88)
Cancer	728 (21.4%)	301 (35.3%)	< 0.001	2.01 (1.71, 2.37)
Depression	16 (0.5%)	17 (2%)	< 0.001	4.32 (2.17, 8.58)
Chronic kidney disease	317 (9.3%)	119 (14%)	< 0.001	1.58 (1.26, 1.98)
Stroke	182 (5.3%)	63 (7.4%)	0.02	1.42 (1.05, 1.91)
Cardiovascular disease	475 (13.9%)	91 (10.7%)	0.01	0.74 (0.58, 0.94)

Table 2 presents the distributions of prescriptions between fall cases and controls. All CNS-active medications, diuretics, calcium channel blockers, diabetic medications, contact laxatives, and proton pump inhibitors correlated significantly (*P* value < 0.05) with an increased risk of falls. Nonsteroidal anti-inflammatory drugs (NSAIDs) may protect against falls.

**Table 2:** Odds ratios (ORs) for falls occurring during hospitalization based on fall-risk- inducing drugs (FRIDS) by class.

Sample size	Non-fall case	Fall case	*P* value	Crude OR (95% CI)
	3408	852		
CNS-active medications				
Opioids				
Non-user	2566 (75.3%)	527 (61.9%)	< 0.001	1 (reference)
User	842 (24.7%)	325 (38.1%)		1.88 (1.60, 2.20)
Antipsychotics				
Non-user	3160 (92.7%)	689 (80.9%)	< 0.001	1 (reference)
User	248 (7.3%)	163 (19.1%)		3.01 (2.43, 3.73)
Anxiolytics				
Non-user	2652 (77.8%)	600 (70.4%)	< 0.001	1 (reference)
User	756 (22.2%)	252 (29.6%)		1.47 (1.25, 1.74)
Hypnotics and sedatives				
Non-user	3096 (90.8%)	731 (85.8%)	< 0.001	1 (reference)
User	312 (9.2%)	121 (14.2%)		1.64 (1.31, 2.06)
Antidepressants				
Non-user	3276 (96.1%)	788 (92.5%)	< 0.001	1 (reference)
User	132 (3.9%)	64 (7.5%)		2.02 (1.48, 2.74)
Antiepileptic				
Non-user	3165 (92.9%)	707 (83%)	< 0.001	1 (reference)
User	243 (7.1%)	145 (17%)		2.67 (2.14, 3.33)
Cardiovascular medications				
Vasodilators used in cardiac diseases				
Non-user	3160 (92.7%)	804 (94.4%)	0.09	1 (reference)
User	248 (7.3%)	48 (5.6%)		0.76 (0.55, 1.05)
Antihypertensives				
Non-user	3287 (96.4%)	816 (95.8%)	0.35	1 (reference)
User	121 (3.6%)	36 (4.2%)		1.2 (0.82, 1.75)
Diuretics				
Non-user	2913 (85.5%)	664 (77.9%)	< 0.001	1 (reference)
User	495 (14.5%)	188 (22.1%)		1.67 (1.38, 2.01)
Beta blocking agents				
Non-user	2735 (80.3%)	677 (79.5%)	0.60	1 (reference)
User	673 (19.7%)	175 (20.5%)		1.05 (0.87, 1.27)
Calcium channel blockers				
Non-user	2805 (82.3%)	670 (78.6%)	0.01	1 (reference)
User	603 (17.7%)	182 (21.4%)		1.26 (1.05, 1.52)
Renin-angiotensin system inhibitors (RAS)				
Non-user	2552 (74.9%)	663 (77.8%)	0.08	1 (reference)
User	856 (25.1%)	189 (22.2%)		0.85 (0.71, 1.02)
Alpha-adrenoreceptor antagonists				
Non-user	3088 (90.6%)	762 (89.4%)	0.30	1 (reference)
User	320 (9.4%)	90 (10.6%)		1.14 (0.89, 1.46)
Digitalis glycosides				
Non-user	3375 (99%)	842 (98.8%)	0.59	1 (reference)
User	33 (1%)	10 (1.2%)		1.21 (0.60, 2.47)
Others				
Anti-Parkinson drugs–anticholinergic agents				
Non-user	3376 (99.1%)	843 (98.9%)	0.75	1 (reference)
User	32 (0.9%)	9 (1.1%)		1.13 (0.54, 2.37)
Anti-Parkinson drugs–dopaminergic agents				
Non-user	3345 (98.2%)	835 (98%)	0.78	1 (reference)
User	63 (1.8%)	17 (2%)		1.08 (0.63, 1.86)
Drugs used in diabetes				
Non-user	2637 (77.4%)	591 (69.4%)	< 0.001	1 (reference)
User	771 (22.6%)	261 (30.6%)		1.51 (1.28, 1.78)
Anti-inflammatory and antirheumatic products, non-steroids (NSAIDs)				
Non-user	2823 (82.8%)	767 (90%)	< 0.001	1 (reference)
User	585 (17.2%)	85 (10%)		0.53 (0.42, 0.68)
Contact laxatives				
Non-user	2321 (68.1%)	488 (57.3%)	< 0.001	1 (reference)
User	1087 (31.9%)	364 (42.7%)		1.59 (1.37, 1.86)
Proton pump inhibitors (vonoprazan excluded)				
Non-user	2256 (66.2%)	515 (60.4%)	0.002	1 (reference)
User	1152 (33.8%)	337 (39.6%)		1.28 (1.10, 1.50)

**Figure 3:**
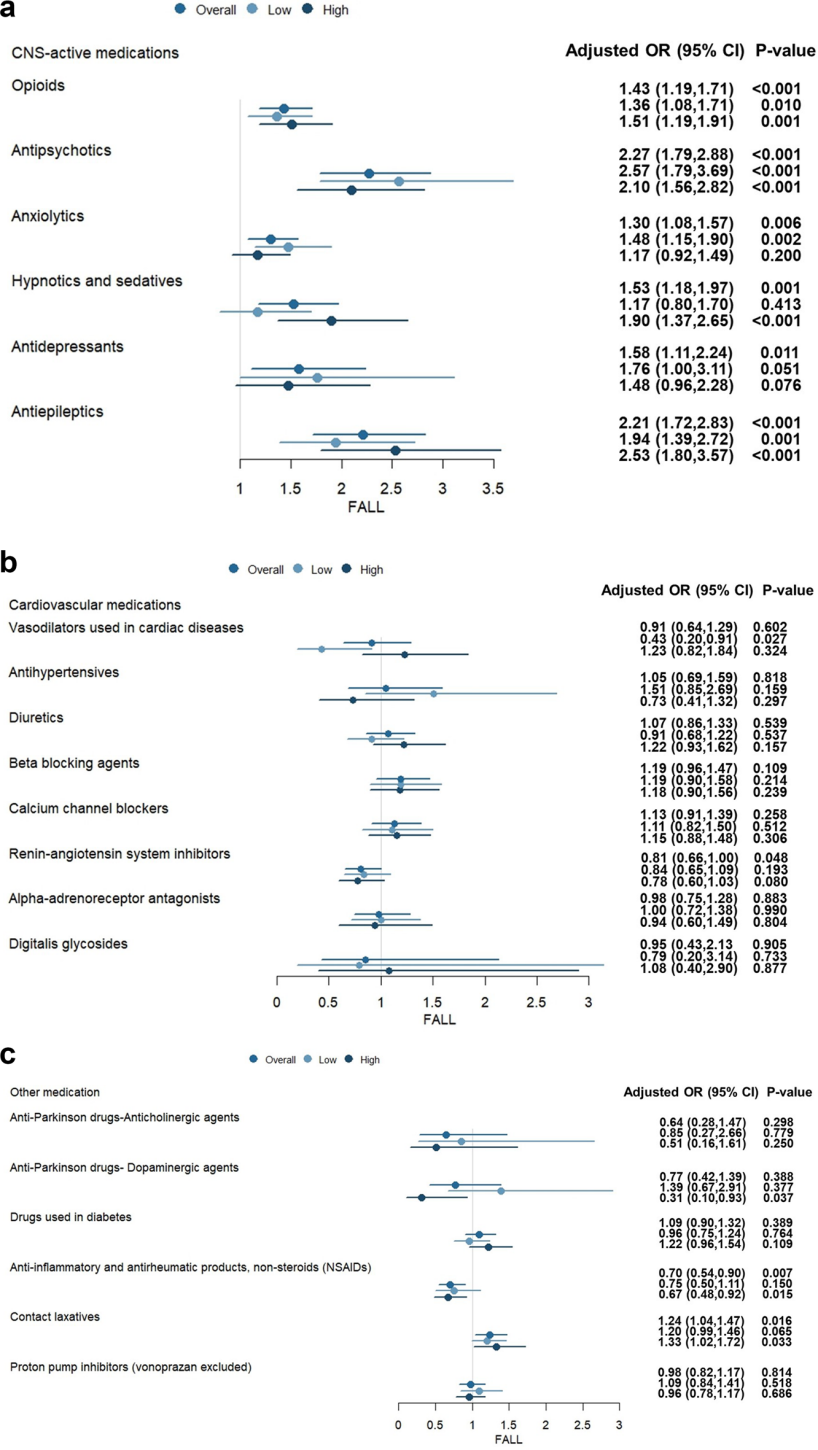
(**a**) Multivariable analysis of fall risk with different doses of fall-risk-increasing drugs during hospitalization: CNS-active medications. (**b**) Multivariable analysis of fall risk with different doses of fall-risk-increasing drugs during hospitalization: Cardiovascular medications. (**c**) Multivariable analysis of fall risk with different doses of fall-risk-increasing drugs during hospitalization: Other drugs.

Patients were divided into high-dose and low-dose groups with the median cut-off point of the defined daily dose (DDD) for each FRID, as depicted in Table 3. Analysis of risk associated with different doses of FRIDs during hospitalization is shown in Fig. 3a and c. Regardless of dose, antipsychotics, antiepileptics, and opioids were associated with higher risk of falls. Other CNS-active medications generally increase the risk of falls, whereas low doses of sedative-hypnotics and standard recommended dose of anxiolytics do not. Among cardiovascular medications, low doses of vasodilators appear to reduce risk of falls. Inpatients taking anti-Parkinson drugs (dopaminergic agents) and NSAIDs, especially high doses, have a lower chance of falls. Finally, contact laxatives are shown to increase the risk of falls.

**Table 3:** The ATC codes and corresponding median cut-off-point of the defined daily dose (DDD) for each fall-risk-inducing drug class.

Drug categories	ATC codes	Median cut-off-point of defined daily dose (DDD)
CNS-active medications		
Opioids	N02A	0.63
Antipsychotics	N05A	0.25
Anxiolytics	N05B	1.00
Hypnotics and sedatives	N05C	1.67
Antidepressants	N06A	1.00
Antiepileptics	N03	1.67
Cardiovascular medications		
Vasodilators used in cardiac diseases	C01D	1.00
Antihypertensives	C02	3.00
Diuretics	C03	10.00
Beta blocking agents	C07	0.52
Calcium channel blockers	C08	3.00
Renin–angiotensin system inhibitors	C09	0.50
Alpha-adrenoreceptor antagonists	G04CA	1.02
Digitalis glycosides	C01AA	1.50
Others		
Anti-Parkinson drugs–anticholinergic agents	N04B	1.30
Anti-Parkinson drugs–dopaminergic agents	N04A	1.27
Drugs used in diabetes	A10	353.38
Anti-inflammatory and antirheumatic products, non-steroids (NSAIDs)	M01A	2.00
Contact laxatives	A06AB	0.65
Proton pump inhibitors (vonoprazan excluded)	A02BC (A02BC08 excluded)	5.33

Table 4 shows the results of multivariable analysis exploring associations between different numbers of FRIDs and falls. The use of more FRIDs is significantly associated with a higher risk of falls. After adjusting for confounders such as baseline characteristics and patient dependency, only the correlation between CNS-active medications and falls remained significant. Falls increase with more diverse CNS-active medications.

**Table 4:** Odds ratios (ORs) of different numbers of Fall-risk-inducing drugs.

	Crude OR (95% CI)	*P* value	Adjusted OR (95% CI)	*P* value
CNS-active medications				
Non-user	1 (reference)		1 (reference)	
User				
1 type used	1.49 (1.24, 1.79)	< 0.001	1.30 (1.07, 1.59)	0.01
2 types used	3.24 (2.62, 4.00)	< 0.001	2.34 (1.85, 2.97)	< 0.001
More than 3 types used	4.45 (3.31, 6.00)	< 0.001	2.63 (1.89, 3.67)	< 0.001
Overall users	2.11 (1.80, 2.48)	< 0.001	1.67 (1.40, 1.99)	< 0.001
Cardiovascular medications				
Non-user	1 (reference)		1 (reference)	
User				
1 type used	1.37 (1.14, 1.64)	0.001	0.95 (0.77, 1.17)	0.62
2 types used	1.27 (1.02, 1.59)	0.04	1.05 (0.81, 1.35)	0.71
More than 3 types used	1.22 (0.96, 1.54)	0.11	1.01 (0.77, 1.33)	0.92
Overall users	1.31 (1.12, 1.52)	0.001	0.99 (0.83, 1.18)	0.90
Others				
Non-user	1 (reference)		1 (reference)	
User				
1 type used	1.27 (1.05, 1.54)	0.01	1.10 (0.89, 1.35)	0.40
2 types used	1.57 (1.27, 1.92)	< 0.001	1.13 (0.89, 1.43)	0.32
More than 3 types used	1.72 (1.28, 2.31)	< 0.001	1.02 (0.74, 1.43)	0.89
Overall users	1.58 (1.33, 1.89)	< 0.001	1.23 (1.01, 1.50)	0.04

The multivariable model was adjusted for CNS-active medications, cardiovascular medications, other medications, and confounders that were significant in linear regression. The confounders included severe illness, education, BMI, number of comorbidities, current smoker, exercise, unstable gait, dizziness, muscle weakness, need for mobility aids, fall history past 1 year, dysphagia, visual impairment, having sleep disorder at admission, dependence level, vital signs within 24-h before the fall (systolic BP, pulse rate, respiratory rate and body temperature), lab data at admission (hemoglobin, creatinine, WBC, platelets, K and Na), and admission diagnosis (anemia, cancer, depression, kidney disease, stroke, and CVD).

## Discussion

To the best of our knowledge, this is the first study to use the defined daily dose (DDD) system to identify FRIDs doses associated with risk of falls. In the present study, the use of more FRIDs was significantly associated with a higher risk of falls. Regardless of dose, antipsychotics, antiepileptics, and opioids were associated with higher risk of falls. However, while CNS-active medications, including antipsychotics, antidepressants, opioids, antiepileptics, and hypnotics, generally increased the risk of falls, low doses of sedative-hypnotics and standard recommended doses of anxiolytics did not. This observation contradicts our initial assumption. Among cardiovascular medications, low doses of vasodilators appeared to reduce risk of falls. Inpatients taking anti-Parkinson drugs (dopaminergic agents) and NSAIDs, especially high doses, had a lower chance of falls.

The present results showing the increased fall risk associated with CNS-active medications (antipsychotics, antidepressants, opioids, antiepileptics, and hypnotics) were consistent with those of several previous studies^7,12,13^. These results suggest that this relationship should be taken into consideration when developing medication management protocols for hospitalized patients. However, because low doses of sedative-hypnotics did not increase fall risk in the present study, we suggest that sleep drugs should be administered in lower doses. Some studies have reported that cardiovascular medications such as antihypertensives may also increase fall risk^14^, whereas the findings of other studies agreed with those of the present study that cardiovascular medications may not increase fall risk^15,16^. For example, although a previous study revealed that vasodilators were responsible for falls^16^, low doses of vasodilators seemed to have a positive effect on falls in the present study.

Furthermore, administering the recommended standard dosage of NSAIDs and prescribing sufficient anti-Parkinson drugs (dopaminergic agents) reduced fall risk in the present study. Although a previous systematic review showed an increased risk of falling due to NSAID use^17^, other studies reached different conclusions^7,18^. In one previous study, opioid recipients had elevated hazard of falls or fractures when compared to NSAID recipients^19^. Thus, using NSAIDs rather than opioids in patient with high fall risk may be a safer choice for pain control. Anti-Parkinson drugs have been reported to be associated with falls in previous studies^14,20^, but this finding may be related to the total burden of FRIDs use in patients treated with anti-Parkinson drugs^21^. Sufficient (standard) dosage of anti-Parkinson drugs (dopaminergic agents rather than anticholinergic agents) improves postural stability and gait, which results in reducing the incidence of falls. Finally, contact laxatives are identified to increase the risk of falls, especially when taken in higher doses. Patients with hypokalemia due to diarrhea may develop muscle weakness, arrhythmia, and fatigue, which may in turn trigger falls.

Several studies found no correlation between the number of FRIDs and fall risk^16,22^. However, in the present study, fall risk increased significantly with the numbers of CNS-active medications, which echoes the recommendation of CNS-active drugs in the Beers Criteria®^23^. Further research is needed to clarify the relationship between the number of FRIDs and fall risk among inpatients.

In the present study, patients with hypokalemia and hyponatremia experienced falls more frequently, a finding that is compatible with those of other studies^24^. Hypokalemia and hyponatremia are the most common electrolyte imbalances in hospitalized patients and are associated with gait disturbances and balance issues^25,26^. Based on a previous trial^24^, even mild hypokalemia (3.0–3.5 mEq/l) and asymptomatic hyponatremia may contribute to falls. Hence, medical professionals are advised to correct the electrolyte imbalances before symptoms onset and to provide patients with fall prevention information.

The heterogeneity of findings in the literature has at least two explanations. First, trials have different study populations, settings, and analysis of adjusted confounding variables. Secondly, the influence of medications dosages was not considered in many previous studies. Physical activity was the only protective personal factor for falls in the present study. One meta-analysis of 40 long-term randomized clinical trials concluded that long-term exercise (≥ 1 year), particularly moderate intensity exercise, significantly reduced falls and fall injuries^27^. Patients should be encouraged to perform multicomponent training with balance exercise at least 2–3 times per week.

The present study has several limitations. First, this is a single-center, retrospective study, which limits the extent to which results can be generalized to other populations. Retrospective study also cannot rule out selection bias. In this study, we only compared prescriptions and laboratory data that were collected within 3 days prior to a fall; other laboratory results and trends may have influenced results. In addition, we use prescription data instead of intake data, which cannot reflect patient compliance. This observational error may slightly influence the results. A mechanism underlying the increased risk of falls could not be proposed based on available data. Finally, because the study was a single-center trial for patients who were admitted to medical or surgical wards, the different patient characteristics should be carefully considered when making generalizations.

## Conclusion

FRIDs, especially CNS-active drugs are significantly associated with fall risk and must be carefully administered in hospitalized patients in order to minimize falls, considering dosage and numbers of such drugs. Sedative-hypnotics can be given in smaller dosages, sufficient dopaminergic anti-Parkinson agents must be prescribed, and NSAIDs use may help reduce the use of opioids. Cardiovascular medications do not seem to be associated with falls as described previously. The effects of vasodilators on falls needs to be further investigated in a well-designed large-scale trial.

## Data Availability

The datasets generated during and/or analyzed during the current study are available from the corresponding author on reasonable request.
